# Adversarial Attack and Defence through Adversarial Training and Feature Fusion for Diabetic Retinopathy Recognition

**DOI:** 10.3390/s21113922

**Published:** 2021-06-07

**Authors:** Sheeba Lal, Saeed Ur Rehman, Jamal Hussain Shah, Talha Meraj, Hafiz Tayyab Rauf, Robertas Damaševičius, Mazin Abed Mohammed, Karrar Hameed Abdulkareem

**Affiliations:** 1 Department of Computer Science, COMSATS University Islamabad, Wah Campus, Wah Cantt 47040, Pakistan; Sheebalal44@gmail.com (S.L.); srehman@ciitwah.edu.pk (S.U.R.); Jamalhussainshah@gmail.com (J.H.S.); talha_cui@ciitwah.edu.pk (T.M.); 2 Department of Computer Science, Faculty of Engineering & Informatics, University of Bradford, Bradford BD7 1DP, UK; 3 Faculty of Applied Mathematics, Silesian University of Technology, 44-100 Gliwice, Poland; 4 College of Computer Science and Information Technology, University of Anbar, Anbar 31001, Iraq; mazinalshujeary@uoanbar.edu.iq; 5 College of Agriculture, Al-Muthanna University, Samawah 66001, Iraq; Khak9784@mu.edu.iq

**Keywords:** diabetic retinopathy, adversarial attack, speckle-noise attack, adversarial training, feature fusion, deep learning

## Abstract

Due to the rapid growth in artificial intelligence (AI) and deep learning (DL) approaches, the security and robustness of the deployed algorithms need to be guaranteed. The security susceptibility of the DL algorithms to adversarial examples has been widely acknowledged. The artificially created examples will lead to different instances negatively identified by the DL models that are humanly considered benign. Practical application in actual physical scenarios with adversarial threats shows their features. Thus, adversarial attacks and defense, including machine learning and its reliability, have drawn growing interest and, in recent years, has been a hot topic of research. We introduce a framework that provides a defensive model against the adversarial speckle-noise attack, the adversarial training, and a feature fusion strategy, which preserves the classification with correct labelling. We evaluate and analyze the adversarial attacks and defenses on the retinal fundus images for the Diabetic Retinopathy recognition problem, which is considered a state-of-the-art endeavor. Results obtained on the retinal fundus images, which are prone to adversarial attacks, are 99% accurate and prove that the proposed defensive model is robust.

## 1. Introduction

A rapidly growing computer vision domain leverages advanced innovation with comprehensive knowledge, while the developed techniques are used for a wide area of applications such as cancer detection [1,2,3], facial expression recognition [4], Parkinson’s disease diagnostics [5,6] and precision agriculture [7,8]. The success of computer vision is due to its more powerful ability to interpret image patterns than the human cognitive visual system. For example, artificial intelligence (AI) based image processing has transformed the field of medical diagnostics in the healthcare domain [9]. Radiomics is an evolving medical imaging field that utilizes a progression of subjective and quantitative examinations of high-throughput image highlights to acquire symptomatic, prescient, or prognostic data from clinical images [10,11]. Image data can take multiple formats, including multi-dimensional data from a 3D scanner or medical scanning devices. Advanced modalities are computed tomography (CT), magnetic resonance imaging (MRI), and nuclear/molecular imaging (which uses biomarkers for in vivo imaging) [12]. Moreover, automated computer vision methods are relevant for health and in-home medical diagnosis [13,14,15].

One such problem successfully addressed by computer vision based diagnostics is the recognition of Diabetic Retinopathy (DR). DR is a chronic disorder that causes blindness in individuals if untreated. A high glucose ratio in the blood causes changes in the retinal microvasculature, resulting in DR, which can lead to a total vision loss. DR is a cause of visual impairment globally that affects nearly 30% of diabetic patients [16]. Early detection of DR through retinal fundus images can avoid possible blindness due to disease. Previous studies have concentrated on the automated early identification of DR by color fundus photography and have produced spectacular classification results [17,18,19].

However, the accuracy and robustness of the deep learning model are frequently plagued by confidentiality of data [20,21]. Minor changes to input images have recently been shown to significantly alter the output of deep learning models [22]. These minor disruptions are an example of adversarial attacks (or adversarial perturbations), which mislead the model, cause it to predict the wrong label, and have drastic consequences for the performance of deep neural network models [23,24]. These models are vulnerable to adversarial examples, which pose a threat in real-world application scenarios [25,26].

Adversarial attacks are categorized as white box, black box and grey box attacks [27]. White-Box (WB) attack has both full information and access to the internal system model. The WB attack can use two iterative methods of the Fast-Gradient Sign Method and the Deep Fool approach, using a set classifier model to reduce its space for searching, and to produce a positive response to unseen adversarial data [28]. The attackers do not know the target model or network, input, and weights in a Black-Box (BB) attack [29]. For BB attacks, reference [30] generated a GenAttack gradient-free optimization algorithm with fewer probes while using Mixed National Institute of Standards and Technology (MNIST) [31], CIFAR-10 [32], and other datasets. Similarly, reference [33] introduced the gradient-based data augmentation technique and substituted ensemble training, which targeted BB attacks on the MNIST and GTSRB datasets with accurate results [34]. In machine learning, the robustness of the adversarial attack detection ability was enhanced by increasing the model capacity with more adversarial training and improved label leaking accuracy [35]. Contractive auto-encoder (CAE) deep neural networks work as a robust model against adversarial examples with high accuracy [36].

Some applications of adversarial attacks using pre-trained deep learning (DL) models in computer vision tasks include, e.g., visual classification [37], textual data system [38], privacy-preserving filter [39], object detector [40], image segmentation [41], natural language processing [42], data fusion [43], hybrid digital watermarking and text document retrieval [44], fingerprint liveness detection [45], person re-identification [46], time series classification [47], human activity recognition [48], face recognition [49], handwritten signature verification [50], and multi-objective reinforcement learning [51].

On the subject of image restoration, noise in an image is crucial. Speckle noise is a type of granular patterning that can be seen in radar coherent images. The Synthetic Aperture Radar image and spatial data both include a lot of speckle noise. In general, SN is the gritty salt-and-pepper pattern seen in radar imaging. It can even be considered a granular ‘noise’ that appears fundamentally in [52] ultrasound, synthetic aperture radar (SAR), active radar, and optical coherence tomography imaging, reducing their quality. Finally, it degrades the performance of critical image processing approaches such as detection, segmentation and classification [4]. A dynamic ultrasound video can be considered three-dimensional (3-D) images with moving parts. It presents a speckle technique for dynamic ultrasound called the 3-D Gabor-based anisotropic diffusion, which has two dimensions in the spatial domain and one in the temporal domain (GAD-3D) [5]. Three test models could be applied to generate synthetic images: radial polar, uniform grid and radial uniform. These synthetic images, which imitate the basic noise features of actual ultrasound images, might be useful for speckle experimentation [53]. Adversarial training is a method of demonstrating and defining the model as a threat by using examples of adversarial situations. In the training phase, it’s also essential to generate and then provide adverse examples from a complete and accurate optimization perspective at least. Whereas this strategy approximates a robust loss, which is precisely the goal we want to achieve, it is frequent to have a lot of the standard loss in the original data points (i.e., gradient measures as well) in that it increases the ‘task standard’ error’s efficiency slightly. Adversarial examples were first prepared using methods such as FGSM, I-FGSM, DeepFool and CW, and then used to train the target model to make it more resilient against an unknown adversarial attack using a diversity adversarial training approach. This technique reduces average attack success rates by 27.2 and 24.3 percent for various adversarial scenarios, while retaining 98.7 and 91.5 percent accuracies for the original data of the MNIST and FashionMNIST datasets, respectively [9]. Features represent the object’s numerical value that expresses the local and global function. The selection of the function features is normally dependent on the problem. Sometimes there are different results according to each feature. In certain cases, the use of a particular feature would be no more successful, so that a successful model is created by a mixing multiple feature. Many people have used different feature fusion techniques because when we fuse the features they have diverse results regarding the research problem. The mixing of characteristics from distinct layers or branches, known as feature fusion, is a common element in current network topologies. This, however, corresponds to iterative attentional feature fusion. On both the CIFAR-100 and ImageNet datasets, our models outperform state-of-the-art networks with fewer layers or parameters [10]. FFU-Net (Feature Fusion U-Net) enhances U-Net from the following characteristic points for diabetic retinopathy lesion segmentation. To decrease spatial loss of the fundus image, the network’s pooling layer is first superseded with a convolutional layer. Then, by fusing contextual channel attention (CCA) models, we combine the multiscale feature fusion (MSFF) block into the encoders, which also enables the network to learn multiscale features efficiently and to enhance the data produced [12]. Diabetic retinopathy is a chronic disorder that cannot be examined properly with normal vision, either aided or unaided, and it is also difficult to predict its density. For the diagnosis and classification of diabetic retinopathy, the key problem occurs when different sensitive sections of the eye, such as retina colors, irregular blood vessels, hard rough exudates, cotton wool spots and different adversarial attacks, are not detected properly. Much work has been done on DR classification and detection with high accuracy, but recently the concept of adversarial attacks has arisen. A small disruption is named an adversarial example/adversarial attack that misleads, with devastating effects, an informed profound neural network model and decreases its accuracy with respect to the correct label. Adversarial attacks against DNN are a serious security obstacle and they decrease accuracy, thus inventing new distance metrics for human perceptual systems and obtaining optimized results via a greedy algorithm [13]. Recently, most work done on adversarial attacks in medical imaging [16], such as stabilized medical image attacks [17], medical image classification [18,19], adversarial learning detecting erroneous diagnoses [20], adversarial heart attacks [21], segmentation of biomedical images [22] and defenses, included binary thresholding [23] using an adversarial attack to evaluate the durability of deep diagnostic models [24] and generative model defense [25] and a critical analysis of antagonistic threats as well as defense mechanisms in physiological computing [26]. Therefore, in this paper, we propose a new Speckle Noise (SN) attack using adversarial image generation, and two defensive methods against these attacks, including defensive adversarial training and feature fusion. The contribution of this research is as follows:

- We evaluate and analyze the adversarial attacks and defenses on retinal fundus images, which is considered a state-of-the-art endeavor.
- We propose a framework that contains a new SN attack, a defensive model against adversarial attacks, the adversarial training (AT), and a feature fusion strategy, which preserves the DR classification result with correct labelling.
- We achieve accurate detection of DR from retinal fundus images using the proposed feature fusion approach.

The remaining paper’s organisation is as follows: Section 2 overviews related work. The proposed method is described in Section 3. Results and analysis are given in Section 4. The research is concluded in Section 5.

## 2. Related Work

During the last few decades, the medical image processing methods help in the early and efficient diagnosis of various severe aliments frequently detected in human beings. Recently, the advanced AI based algorithms have attained great importance with high accuracy in the classification of medical images and the detection of diseases in the medical field with productive results. Two-fold detection of DR using morphological procedures was introduced [54], which detects microaneurysms, exudate, blood vessels and second severity of its type using Support Vector Machine (SVM), but through adversarial attacks, their credibility has decreased. Deep radiomics performs well in medical imaging, but accuracy has deteriorated, and the incorrect label is based on minor disturbances (SP). In this regard, reference [55] introduced two novel attacks—Bracketed Exposure Fusion (BEF) and Convolution Bracketed Exposure Fusion (CBEF)—based on component-wise multiplicative fusion and element-wise convolutional for the detection of diabetic retinopathy (DR) by using the Eyepacs Dataset with high-quality images and transferal rates.

Universal perturbations attacks (UPA), which used iterative algorithms for targeted and non-targeted attacks, were proposed by [56], and achieved 80% accuracy in classification. Reference [57] presented two lightweight techniques, which used local perturbation and universal attacks. The sequential decision method for fixing the image reconstruction model is implemented using reinforcement learning [58]. The adversarial data augmentation approach proposed by [59] for medical image segmentation was designed for deep neural network (DNN) model training induced by a shared type of artifact in magnetic resonance imaging (MRI).

The adversarial augmentation approach was proposed in [60], which was used to generalize the model. Project gradient descent (PGD) or adverse synthetic nodule and adverse perturbation noise work detected the lung by false positive reduction (FPR). For malignancy prediction of lung nodules, reference [61] introduced an adversarial attack deep neural network ensemble methodology for classification using FGMS and 1-pixel attack, achieving 82.27% and 81.43% accuracy. The authors proposed a DL-based encryption and a decryption network (DLEDNet) [62] using an X-ray image dataset through region of interest (ROI) segmentation in an encrypted medical image. In medical imaging for adversarial training, reference [63] developed transfer learning and a self-supervision based procedure for adversarial training for pneumonia classification of X-ray images and MRI segmentation using PGD and fast gradient methods. The detailed comparison of recent related works with their dataset description is presented in Table 1.

### Defenses against Adversarial Attacks

Reference [65] proposed defense against two groups: feature-level interpretation and model-level interpretation, input denoising, and model robustification. Two image transformations, Discrete Wavelet Transform (DWT) and Sine Transform (ST), were presented by [66] for classifying features with a SVM classifier. Some techniques demonstrated by [67] included dimensionality reduction, a characterization of the adversarial region, and combining input discretization with adversarial training. Activation transformations for the best and most robust defense against these attacks were also considered. Meng and Chen [68] proposed the MagNet DNN classifier, which performs classification and reformer networks against adversarial examples (AEs) on the manifold and standard examples. Another defense method against AEs is Hilbert-based Generative defense introduced by Bai et al. [69], which worked as a pixel CNN on different dimensions and improved their results more accurately. For weak or small adversarial attacks, for example, in crafted attacks in DNN background class, a training process works as a defense [70]. Reference [71] introduced pro-trace vectorization algorithms defense against adversarial attacks on the MNIST digits dataset. The defense obfuscated gradient-based approach [72] gives false sensitive security and was tested on different nine attacks with accurate results. Another defense for adversarial attacks on MNIST digits dataset proposed [64] a Mary EAD elastic-net attack with minimum distortion. Reference [73] suggested that the defense of the adversarial attack on a fuzzy unique image transformation (FUIT) method used down-sampling while using a chest X-ray and a CT image dataset for the diagnosis of COVID19 through a DNN model.

## 3. Methodology

**Figure 1:**
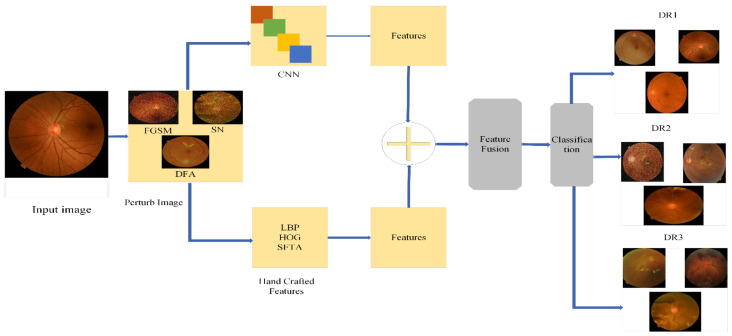
Block diagram of the proposed system.

The proposed methodology performs DR classification using original and perturbed images, and the accuracy is preserved by including different adversarial attacks, such as FGSM and SN DF, in which a speckle noise (SN) attack is a novel attack. The presence of these attacks decreased the model’s credibility and the wrong classification was made. To overcome this problem, adversarial training and feature fusion were proposed as two defensive strategies against adversarial attacks. We performed four distinct training sessions with fine-tuned transfer learning utilizing the Darknet53 model and outcomes in adversarial training. For robust results, we integrated deep and handcrafted features in feature fusion. The handcrafted features included HOG, FHOG(Sv) SFTA FST(Tv), LBP, FLBP(Uv) and FDARK53(xv), which increased the accuracy. All primary steps are shown in Figure 1 and explained in the subsections below.

In this proposed defense model block diagram, we used both the original and perturbed images dataset prepared by using a data augment technique and then resizing the data images into 224×224×3. Perturbed images generated through FGSM and SN/MN and DEEP FOOL attacks were applied to the dataset and three types of perturbed images were generated. Adversarial attacks decreased the accuracy with abrupt changes and misclassified the model. To overcome this problem, we proposed the feature fusion defense, in which we combined the deep features using Drknet53 features extracted by applying fine-tuned transfer learning In the proposed model, we use conventional extraction methods of LBP, HOG, and SFTA, which extract robust, immutable features to image, translate and display. These attributes are based on appearance and composition, an essential component in defining the characteristics of the images of an individual’s eyes. This feature fusion works as a robust model against these adversaries, which fooled our network and maintained accuracy with high precision.

### 3.1. Data Augmentation and Pre Processing

A dataset with 1000 instances from Kaggle has been taken with three diabetic retinopathy classes: DR1, DR2, and DR3 fundus images with minor, moderate and severe conditions. We used a data augmentation approach that involved flipping and flopping at various rotations to construct a new dataset with 6497 images. Regional resolution is the lowest in the retinal images. Since the original dataset images are 2592×1728 too large and complicated to be processed further for this dimension, the time spent in the RGB (red/green/blue) channel to produce adversary attacks is reduced, and the images are resized to 224×224×3.

### 3.2. Transfer Learning

Fine-tuned transfer learning is applied; it involves using the features learnt from one issue and a new related concern. The fine-tuning of the DarkNet-53 [76] model involves unfreezing the whole or part of these model structures and re-training it with a meagre learning rate on the new results. This could lead to significant changes by adapting the pre-trained functionality to the new data gradually. Fine-tuning is an interesting activity that entails unfreezing the entire model (or a section of it) and retraining it on new data with a very modest learning rate. By incrementally modifying the pretrained features to the new data, this has the capability for considerable improvements. Using a fully convolutional neural network, a multi-source adversarial transfer learning approach enables the development of a feature representation glucose prediction for diabetic persons. The evaluation is carried out by examining several transfer scenarios using three datasets with considerable inter and intra variation [29]. COVID-19 is diagnosed using Distant Domain Transfer Learning (DDTL) [30]. COVID-19 detection used fine-tuned convolutional neural networks and confined in chest X-ray images [31]. We used fine-tuned transfer learning in our proposed work, which creates a basic model and loads pre-trained weights into it. The FC layer was removed from DarkNet53 and was replaced with the ’new classoutput’ layer. Some convolutions layers were frozen. Various parameters and loss functions were optimized. It was run with a new dataset and the output of one (or more) layers was recorded from the basic model. Feature extraction is the term for this process, using the output as the basis for a new, more compact model.

### 3.3. Perturbed /Adversarial Image Generation

Many Deep Neural Network (DNN) adversaries have recently been revealed as the source of defects. In addition to the research entry, these disruptions are small and unnoticeable to humans, but the output of the network becomes unpredictable.

#### 3.3.1. Fast Gradient Sign Method (FGSM) Attack Image Generation

**Figure 2:**
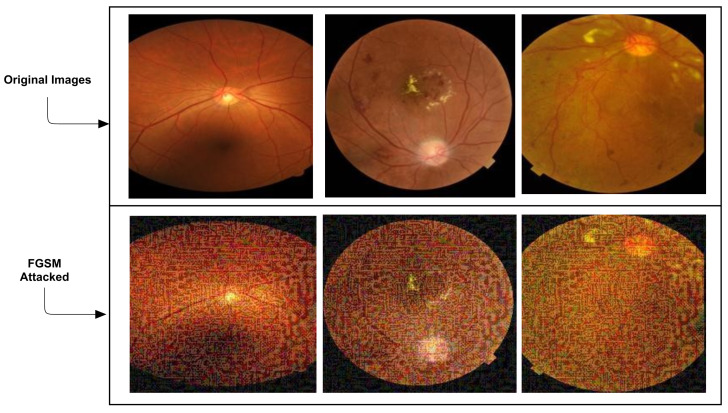
Addition of FGSM Attacks. The first row shows the original images, while the second row represents the FGSM attacked images that mislead the model.

The attack changes the entry data to optimize the loss based on the same back-propagated gradients rather than mitigating loss. In other terms, the attack uses the malfunction gradient to change the data to increase the loss [77]. FGSM is based on the standard networks’ principle implementing the gradient descent to set a minimum loss point. We can maximize the loss by only adding a small perturbation in the case of following the sign of gradient descent, described as:

(1) $$I^{prt}=I+\in *\Delta \left(\partial_{y\;}\;l(I,Z_{tl}\right),$$

where *I* is an original image *Iprt is* adversarial image, ∈ is a multiplier to guarantee the perturbations are minor, ∂ are model parameters, l is the classification loss function and *Ztl is* a true label for original input I. Examples of the FGSM attacked images, which mislead the model, are presented in Figure 2.

#### 3.3.2. Speckle Noise (SN) Attack Image Generation

Frequently known as multiplicative noise or speckle noise (MN/SN), multiplicative noise is less frequent than additive white Gaussian noise (AWGN). However, it is widely used in incoherent image acquisition, including radar and synthetic sonar depth of field, and primarily for medical imaging, using ultrasound and laser imaging techniques. The systematic interference of waves reflected from several primitive scatterers causes speckle to appear in synthetic aperture radar images. This generates pixel-to-pixel intensity variance, which appears as granular noise in SAR images [4]. Because of the system’s function, noise is more complex and challenging to cope with:

1. Each pixel of the original image is composed of noise components.
2. The noise of speckle is not usually distributed and similar to the Rayleigh and Gamma distributions described below:

(2) S=(I+n×I),

where *n* is random noise with a mean of 0 and variance of *s* is uniformly distributed, *s* is set to 0.50 by default. The value of *s* might be anywhere between 0 and 1. The mean and variance parameters for the gaussian’, ssian’, 0.50 by localvar’ noise types are always supplied as if the image were of class double in the range, with 0 indicating no noise and 1 indicating a completely noisy image (0,1).

The medical images are significantly degraded because of these images:

1. Noise is unavoidable in the process of data acquisition.
2. Low contrast due to the variations of lighting and a variety of other causes.
3. Random pixel values for individual pixels of an image can be created by multiplying speckle-noise.

**Figure 3:**
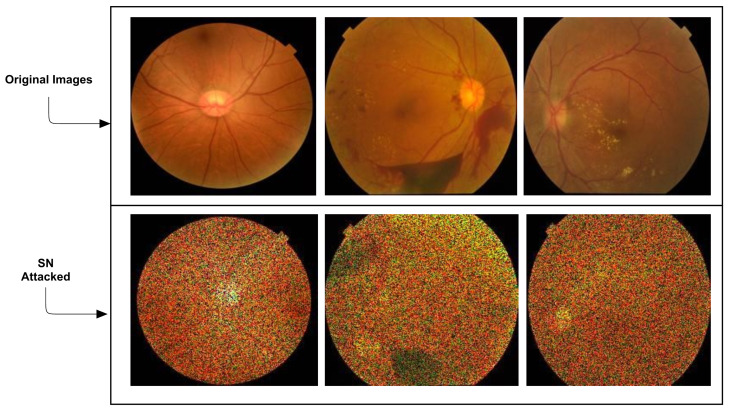
Addition of FGSM attacks. The first row shows the original images, while the second row represents the SN attacked images.

The addition of SN attacks is given in Figure 3.

#### 3.3.3. Deep Fool Attack Generation

Deep Fool (DF) is an opposing attack aimed at taking an example of the closest boundary. In contrast to rough extrapolations of an optimal distributive vector generated by FGSM, according to the authors, this method produced a subtle disturbance. The DF attack uses one loss gradient in *l* (f(k) and *y*) as follows [78].

(3) $$\Delta \left(I;b^{\;}\right)=:min_{c}∥c∥subjecttob^{\;}\left(I+c\right)\neq b^{\;}\left(I\right).$$

Here, *I* is an original image, b is estimated label, *c* is minimal perturbation.

Deep-Fool describes optimization for a two-class problem as follows. Deep-Fool can have a simple solution for multi-class problems if the classifier is one-vs-all. Here, we mean the classification system of one-vs-all, taking into account two-class concerns, where *n* are the number of classes which are also the number of discrimination-related functions. However, the one-vs-all method does not apply to a linear machine because one-vs-all essentially manages a series of separating hyperplanes while one-vs-all does not apply. In contrast to rough extrapolations of optimal distributive vector generated by FGSM, according to the authors this method produced stubble disturbance.

**Figure 4:**
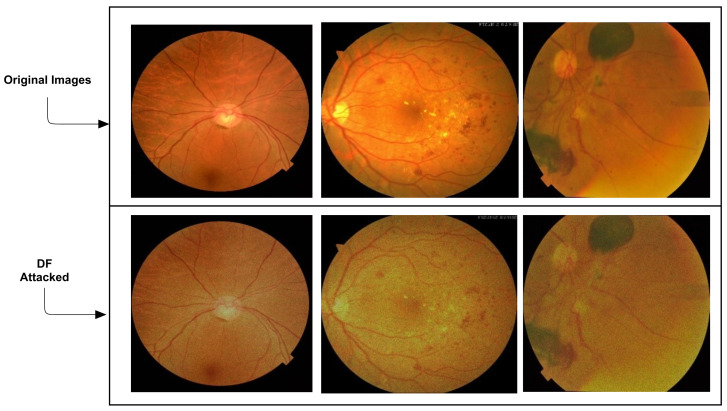
Addition of DF attacks. The first row shows the original images, while the second row indicates the DF attacked images.

The addition of DF attacks is given in Figure 4.

### 3.4. Proposed Defense against Adversarial Attacks

#### 3.4.1. Adversarial Training (AT)

In the proposed defense method, we have done four adversarial training (AT) on a dataset. In the first training session, we take half of the original data and half of our FGSM adversarial images of three classes: DR1, DR2, DR3, and trained a new prepared dataset from scratch using deep network DarkNet-53 model through fine-tuning and transfer learning. The proposed model extracted the features and made predictions, which were further checked through testing. The testing is performed on the newly trained dataset using original and perturbed images. The accuracy measure increases when the testing is performed on originally trained data in the second adversarial training. In the second adversarial training, two parts of the dataset, images, were included in which half of images of the original dataset and half of speckle-noise (SN) attacks images data set of every class included DR1, DR2, and DR3. In the third training, images were included in which half of images of the original dataset and half of Deepfool (DF) attacks images data set of every class included DR1, DR2, and DR3.

We equally divided the whole data into four parts in this training in which original, the FGSM, SN, DF attacked images were included according to each class data images DR1, DR2, and DR3. This defense is more robust than the first one, because in this training, more data is given, and classifier learns to work best, and model fooling chances are less when compared to the first one. Through the testing process, we check can the defensive model accuracy and robustness.

1. **Training 1**: original + FGSM attacks images (AT1)
2. **Training 2**: original + SN attacks images (AT2)
3. **Training 3**: original + DF attacks images (AT3)
4. **Training 4**: original + FGSM + DF images (MAT)

**Figure 5:**
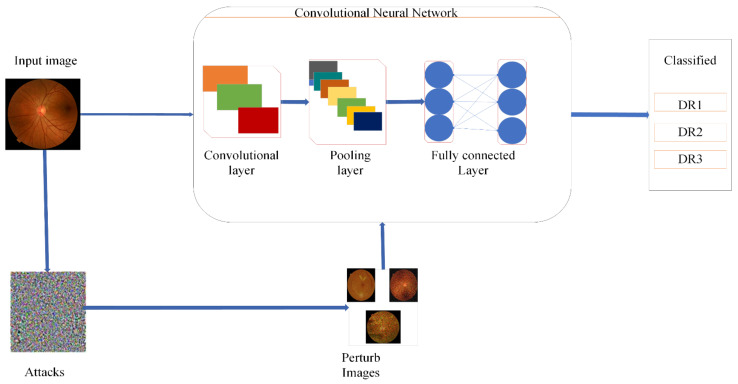
Illustration of the adversarial training process.

Adversarial training architecture of all types of data sets is given in Figure 5 [34,35,52].

#### 3.4.2. Feature Extraction and Feature Fusion Defense

In the proposed method, we use conventional extraction methods of Local Binary Pattern (LBP) [79], Histogram Oriented Gradient (HOG) [80], and Segmentation Based Fractal Texture Analysis (SFTA) [81], which extract robust, immutable features to image, translate, and display. These attributes are based on appearance and composition, which are essential components in defining the characteristics of the images of an individual’s eyes.

#### 3.4.3. Local Binary Pattern (LBP)

LBP is a primary but very effective method that labels the image pixels by threshing every pixel region and takes the output as a binary number. LBPs is a part of the computer vision classification visual descriptor. The LBP descriptor by its specifications, represents the input image. For capturing images such as boundaries, spots, and flat regions used it. Feature vector F_LBP is calculated as:

(4) $$LBP\left(x,y\right)=\sum_{P=0}^{P-1}t\left(g_{np}-g_{cp}\right)2^{P}$$

where $g_{np}$ is the intensity of neighboring pixel, and $g_{cp}$ is the intensity of the central pixel $t\left(x\right)$, which can be defined as:

(5) $$t\left(x\right)=\left\{\begin{array}{c} 1\;if\;x>0\\ 0\;if\;x<0 \end{array}.\right.$$

#### 3.4.4. Histogram Oriented Gradient (HOG)

The strategy calculates gradient orientation instances in the located sections of an image. This approach is close to that of histograms of edge orientations, scale-invariant descriptors, and shape contexts, but differs in that this method is measured on a dense grid of continuously adjacent cells using local contrast normalization, which overlap for enhanced precision. The number of pixels is specified for each cell, and the histogram of the gradients is then computed for each cell. The Laplacian and Sobel operator give *u* the direction of HOG. The gradient of *f* is given as a column vector for a function f(x,y) at the coordinates (x,y):

(6) $$\nabla f=\left[\begin{array}{c} G_{x}\\ G_{y} \end{array}\right]=\left[\begin{array}{c} \frac{\partial f}{\partial x}\\ \frac{\partial f}{\partial y} \end{array}\right]$$

The magnitude of this vector is given by:

(7) ∇f=mag(∇f)

(8) $$f\left(x,y\right)={tan}^{-1}\left(f_{y}\left(x,y\right)/f_{x}\left(x,y\right)\right).$$

Feature vector $F_{HOG}$ is calculated through it.

#### 3.4.5. Segmentation Based Fractal Texture Analysis (SFTA)

SFTA is an active texture-based extraction method. It gives a set of reliable features of an individual not overemphasised by scaling, rotation, and translation complications. The SFTA characteristics are immune to the “noise” effects of the image. Feature vector$\;F_{ST}$ is calculated through it. The SFTA functionality is sensitive to the image impact of “noise” SFTA transforms an image of an individual into a binary as an input (referred to below equations):

(9) $$i_{bny}\left(k,l\right)=1\;if\;r_{lw}<i\left(k,l\right)\leq r_{up}\;\;\;\;\;\;\;\;\;\;\;\;0\;,\;\;otherwise$$

where $i_{bny}\left(k,l\right)$ is a resulting binary image, an input image is denoted by i(k,l) and $r_{lw}$ and $r_{up}$ upper and lower threshold values. SFTA utilizes a threshold value by multimodal Otsu algorithm [82]. SFTA then calculates the binary fractal calculation border area.

(10) $$\nabla \left(k,l\right)=1\;if\;\exists \left(k^{\prime },l^{\prime }\right)\epsilon N_{8}\left[\left(k,l\right)\right.]$$

$$l_{b}\left(k^{\prime },l^{\prime }\right)=0\;$$

$$l_{b}\left(k,l\right)=1,\;\;\;\;\;\;\;\;\;\;\;\;\;\;\;\;\;\;\;\;\;\;\;\;\;\;\;\;\;\;\;\;\;\;\;\;\;\;\;\;\;\;\;\;\;\;\;\;\;\;\;\;\;\;\;0,otherwise$$

where Δ(k,l)is consequential boundary image,$\;l_{b}\left(k,l\right)$ is a binary image and N[(k,l)] connected pixel value Δ(k,l)has value 1, if the corresponding $\;l_{b}\left(k,l\right)$ has value 1, and otherwise 0. SFTA generates an invariant image vector for scaling, rotation, or translation.

### 3.5. Deep Feature Extraction

The DarkNet-53 network model contains 53 layers, including input and output layers. Transfer learning is used through DarkNet-53. There are 184 layers in DarkNet-53, including one input layer, 53 convolutions layers, 53 Batch Normalization (BN) layers, 52 Leaky ReLU, 23 Addition, 1 Global Average pooling layer, and 1 classification output softmax layer. The image size of the input of a network is 256×256. The detector module consists of several Conv layers clustered in blocks, up-sampling layers, and three Conv layers, which are linearly activated and allow detections at three different scales. There is no max-pooling layer present in DarkNet-53. Instead, it uses BN and leaky RELU layers for every convolution step.For deep features extraction using DNN, we used trained Darknet53 from starch to classify the different stages of diabetic retinopathy which included DR1, DR2, and DR3.Darknet53 is used in image processing many tasks included object detection, real-time object detection YOLO, image classification, segmentation, model compression, fruit classification [52] etc. In medical imaging darknet53 used for detection of covid-19 [34] computed aided covid-19 detection [36] for MRI scan brain tumor data augmentation [35] YOLO V3 has been used to identify red lesions in retinal fundus images [37]. smart medical autonomous distributed system for diagnosis [38], melanoma detection [39].

#### Feature Fusion (FF)

We have fused the hand-crafted features with deep features to obtain a single vector. A serial feature fusion approach is used in the proposed method. The feature vector obtained is more efficient, since it includes additional information than these, which we obtain by using a single extractor procedure.

Three function vectors like the HOG, SFTA, and LBP are allowed by the suggested method $F_{HOG}\left(S_{v}\right)$, $\;F_{ST}\left(T_{v}\right)$, $F_{LBP}\left(U_{v}\right)\;$ deep characteristic allows for the DarkNet-53 $F_{DARK53}\left(x_{v}\right)$.I×J is the dimensions of it. The classification os performed using the following equation:

(11) $$F_{i*j=}\left(S_{v}+T_{v}+U_{v}+F_{Darknet53}\right).\;$$

## 4. Results and Discussion

In this section, the experimental results are presented. Table 2 shows the experimental results obtained on the attacks’ images, three different attacked images, fast gradient sign method (FGSM) attack images, speckle noise (SN) attack images, and deep fool (DF) attacked images. The unexpected changes in accuracy results occur in this part of the experimentation.

Table 2 shows abrupt changes in the prediction, when testing the attacked images with the originally trained network; they wrongly predicted their classes with maximum accuracy all wrongly predicted values highlighted in this tables, which shows wrong labels of each class. When FGSM attacked images of class DR1 were tested, it predicted as belonging to class DR2 with 93.01% accuracy, while they are from class DR1 same as for other classes of DR, class DR3 attacked images were misclassified. When speckle-noise attacked images of class DR2 were tested, they are categorised as class DR3 with 79.09%. The other two classes images wrongly predicted with a high precision rate due to a speckle-noise attack. For DF, when class DR3 image was tested, it was predicted into class DR2 with 99.75% accuracy, while DR1, DR2 classes images were also wrongly labelled.

Table 3 shows the testing accuracy of FGSM attack Dataset images, SN attack images, and DF attack images (Adversarial Training AT1). In the first training session, we take a half of the original data and a half of our FGSM adversarial images of three classes: DR1, DR2, DR3, and trained a new dataset from scratch using DarkNet-53 through fine-tuning transfer learning. The model extracted the features and made predictions which checked through testing. When testing performed on this newly trained network using original and perturbed images, the accuracy measure increase compared to already testing performed on the trained network initially.

When the adversarial training AT1 network is tested through the FGSM attack images, the results are presented in Table 2, in which the FGSM attacked image was misclassified through maximum 93.01% is classified into DR2 class after this training correctly labelled with 88.86% in DR1 class, and 0% chance that it belongs to DR2 class same as in the other class images. In the SN and DF attacked images, some images were correctly labelled with 40% and 64.51% accuracy.

Table 4 shows the testing accuracy of the FGSM attacks dataset images, SN attacks images, and DF attacks images with Adversarial Training AT2. In the second adversarial training, two parts of the dataset, images were included in which a half of images of the original dataset and half of the speckle noise (SN) attacks images data set of every class included DR1, DR2, and DR3 classes, and network trained using fine-tuned transfer learning and testing performed results are shown in Table 4.

When adversarial training AT2 network was tested through attack images, the detected anomaly resolved all the SN attacked images classified with high 98.02%, 88.66%, 94.07% accuracy and the other two attacked images are also correctly classified.

Table 5 showed testing accuracy of FGSM attacks images, SN attack images, and DF attack images with Adversarial Training AT3. In the third training, images were included in which a half of images of the original dataset and a half of Deep-fool (DF) attacks image data set of every class included DR1, DR2, and DR3 classes. The network trained using fine-tuned transfer learning and testing performed on all types of attacked images and the result is shown in Table 5.

When adversarial training AT3 network tested through attack images, the anomaly present in the previous tables was resolved. Most of the FGSM and SN attacked images were correctly labeled with a maximum accuracy, and all the DF attacked images were correctly classified with 82.97%, 99.96%, and 100% accuracy.

Table 6 shows the testing accuracy of the FGSM attack dataset images, SN attack images, and DF attack images with Mixed Adversarial Training (MAT). We equally divided the whole data into four parts in which the original FGSM, SN, and DF attacked images were included according to each class of images DR1, DR2, and DR3. This defense is more robust than the previous scenarios, because more data is given in this training, and classifier learns to work best, and model fooling chances are less. Through the testing process, we check the accuracy and robustness of the defensive model.

Through mixed adversarial training (MAT), the results of MAT were obtained in which the majority or attacked images were correctly classified with high accuracy and precision, and the trained model became most robust. To incorporate and deal with the adversarial attacks, we performed the adversarial training on mixed data, and the testing results revealed that almost half of the labels were predicted correctly. Moreover, we also used the original, FGSM, SN, and DF datasets for adversarial training, which performs efficiently and labels the majority of the labels correctly. The summary of the defensive proposed model is presented in Table 7.

### Feature Extraction and Feature Fusion Defense

**Figure 6:**
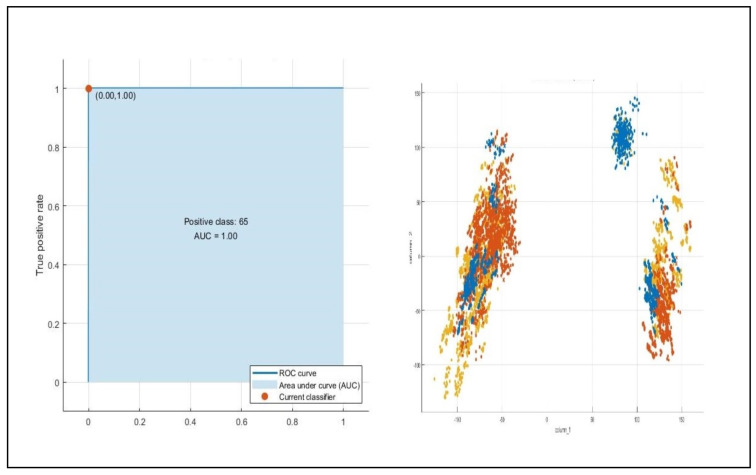
ROC curve (**left**) and fusion scatter plot (**right**).

A serial feature fusion approach is used in the proposed method. Three function vectors from HOG, SFTA, and LBP methods are allowed by the suggested method $F_{HOG}\left(S_{v}\right)$, $\;F_{ST}\left(T_{v}\right)$, $F_{LBP}\left(U_{v}\right)\;$deep characteristic allows for the DarkNet-53 $F_{DARK53}\left(x_{v}\right)$.I×J is the dimensions of it. Our proposed feature fusion method achieved robust results on adversary images and classified correctly. The results obtained using feature fusion approaches are given in Table 8 and Table 9. Furthermore, the ROC curve and fusion scatter plots for the proposed method are visualized in Figure 6.

## 5. Conclusions

The attacks against artificial intelligence models can decrease model performance. We have proposed an adversarial training based defense against adverse disruptions in order to address this problem. The method improvement includes not simply an advanced understanding of image processing techniques, but also needs essential medical input, including expert knowledge related to diabetic retinopathy and its screening procedure, in addition to the eye fundus photography process.

To mitigate the impact of adversarial attacks, we have executed different kinds of adversarial training, through which the adverse effect is reduced, and with which the model cannot become fooled when compared to existing models. Results obtained are 92% correct and prove that the proposed defensive model is robust. Another defense model based on feature fusion was also proposed for adversarial attacks, in which deep and handcrafted features were fused, including the DarkNet-53 deep features and LBP, HOG, SFTA features, and the accuracy was increased by 99.9%.

## Figures and Tables

**Table 1:** Comparison of recent related works with their datasets.

Reference	Methodology	Dataset	Evaluation Measures	Results
[54]	Morphological operation	DiaretDB	SVM classifier	Mild severity DR detection and classification
[55]	Bracketed Exposure Fusion (BEF) and Convolution Bracketed Exposure Fusion (CBEF) Attacks	Eyepacs	Component-wise multiplicative fusion and element-wise convolutional	DR detection
[56]	Iterative algorithms for universal perturbations attacks (UPA)	Multiple datasets	Classification of targeted and non-targeted UPA attacks	80% Accuracy
[57]	Local perturbation and universal attacks,	Cityscapes	Noise function and Gradient of pixels	Image Segmentation
[58]	Reinforcement learning, Markov Decision Process	MRI single-coil knee dataset	MSE, NMSE, SSIM and PSNR	MRI phase-encoding sampling
[64]	Adversarial training by modelling intensity inhomogeneities	Automated Cardiac Diagnosis Challenge (ACDC)	Low-shot learning, learning from limited population	Semantic features for cardiac image segmentation
[60]	Projected gradient descent (PGD), adverse synthetic nodule and adverse perturbation	CT data	False positive reduction rate	Lung nodule detection and prediction of lung by false positive reduction (FPR)
[61]	Fast Gradient Sign Method (FGSM) and one-pixel attacks	National Lung Screening Trial (NLST) dataset	Ensemble-based classification	Malignancy prediction of lung nodules. 1-pixel attack with 82.27% and 81.43%
[62]	Cycle-generative adversarial network (Cycle-GAN)	Chest X-ray data set	X-ray dataset through ROI (region of interest)	Encrypting and decrypting the medical image through DeepEDN
[63]	Self-supervised transfer learning combined with adversarial training	Chest X-rays, and segmentation of MRI images.	MRI segmentation using two PGD, and fast gradient single method	Pneumonia classification of x-ray images and MRI segmentation
[65]	Untargeted vs Targeted Attack, One-Shot vs Iterative Attack	Fashion-MNIST dataset	Feature-level interpretation and model-level interpretation	Defensive graph-based models, causal models generated
[66]	Discrete Wavelet Transform and Discrete Sine Transform	Object database (validation set of ImageNet) and face recognition (MBGC)	SVM Classifier	Defense through which adversarial perturbation can be neutralized
[67]	Dimensionality reduction, a characterization of the adversarial region,	Multiple dataset	Combining input discretization with adversarial training	Activation transformations for the best and robust defense against these attacks
[68]	MagNet with Randomization	Adversarial examples (AEs) on a manifold and normal examples.	MagNet DNN classifier	3% higher than simple MagNet.
[69]	Hilbert-based Generative pixel CNN Hilbert-based PixelDefend (HPD)	Adversarial examples (AEs)	Ensemble of Hilbert curve with different orientations.	PixelDefend mapping pixels from 2-D to 1-D.
[70]	Crafts attacks, Background class image classification training	EMNIST Dataset	Weak or small adversarial attacks samples based	Constructing background images between the key classes and artificially expanding the background data
[71]	Protrace vectorization algorithms	MNIST handwritten digits dataset	In high-dimensional color image space, simple image tracing may not yield compact and interpretable elements.	the vector images are resolution-independent, one could rasterize them back into much smaller-sized images.
[72]	Obfuscated Gradients, iterative optimization-based attacks,	ICLR 2018	False sensitive security	Prevent gradient descent-based attacks) for perceived robustness
[64]	Mary EAD elastic-net attack with L∞	MNIST digits dataset	local first-order information, Minimum distortion	EAD is able to outperform PGD in transferring in the targeted case.
[73]	Fuzzy Unique Image Transformation (FUIT)	Chest x-ray and CT image dataset	that downsamples the image pixels into an interval.	Diagnosis of COVID-19 through DNN model.
[74]	Feature Squeezing	MNIST, CIFAR-10, ImageNet	Joint detection with multiple squeezers, adversarial adaptation	Color depth reduction, median smoothing. non local smoothing
[75]	Perceptual hash	CIFAR-10	JSMA gradient-based attack, One Pixel Attack is an evolutionary-based attack	White-box attack success rate 36.3%, and in black-box attack 72.8%

**Table 2:** Testing of Original Training Network with Attack Images.

Original Class Label	Attacks Applied	Predicted Label After Attack	Accuracy with Class DR1 (%)	Accuracy with class DR2 (%)	Accuracy with Class DR3 (%)
DR1	FGSM	DR2	0	93.01	6.99
DR2	FGSM	DR1	81.71	0	18.29
DR3	FGSM	DR2	0	91.09	8.91
DR1	SN	DR2	12.27	87.98	0
DR2	SN	DR3	10.09	10.82	79.09
DR3	SN	DR1	89.82	10.18	0
DR1	DF	DR2	0	100	0
DR2	DF	DR1	82	17.59	0.41
DR3	DF	DR2	0	99.75	0.41

**Table 3:** Testing of Adversarial Training AT1 Network with Attack Images.

Original Class Label	Attacks Applied	Predicted Label After Attack	Accuracy with Class DR1 (%)	Accuracy with Class DR2 (%)	Accuracy with Class DR3 (%)
DR1	FGSM	DR1	88.86	0	11.14
DR2	FGSM	DR2	20	72.07	7.93
DR3	FGSM	DR3	10.01	8.94	81.05
DR1	SN	DR2	40	57.23	2.77
DR2	SN	DR1	70	0	30
DR3	SN	DR3	0	0	40
DR1	DF	DR3	0	40.97	58.50
DR2	DF	DR1	89.79	0.21	10.0
DR3	DF	DR3	0	35.32	64.51

**Table 4:** Testing of Adversarial Training AT2 Network with Attack Images.

Original Class Label	Attacks Applied	Predicted Label After Attack	Accuracy with Class DR1 (%)	Accuracy with Class DR2 (%)	Accuracy with Class DR3 (%)
DR1	FGSM	DR3	2.71	20.3	76.98
DR2	FGSM	DR2	0	62.37	37.26
DR3	FGSM	DR1	81.34	4.7	13.96
DR1	SN	DR1	98.02	1.98	0
DR2	SN	DR2	0.02	88.66	11.32
DR3	SN	DR3	0	5.98	94.07
DR1	DF	DR2	14.62	71.8	13.58
DR2	DF	DR3	0	10.15	89.95
DR3	DF	DR1	82.75	1.8	15.45

**Table 5:** Testing of Adversarial Training AT3 Network with Attack Images.

Original Class Label	Attacks Applied	Predicted Label After Attack	Accuracy with Class DR1 (%)	Accuracy with Class DR2 (%)	Accuracy with Class DR3 (%)
DR1	FGSM	DR1	68.05	20.97	20.09
DR2	FGSM	DR3	29.91	10.91	50.09
DR3	FGSM	DR3	3.90	35.32	74.51
DR1	SN	DR3	39.91	0	60.09
DR2	SN	DR2	9.87	90.02	0.01
DR3	SN	DR3	0	45.32	54.51
DR1	DF	DR1	82.97	17.02	0
DR2	DF	DR2	0.04	99.96	0
DR3	DF	DR3	0	0	100

**Table 6:** Testing of Mixed Adversarial Training MAT Network with Attack Images.

Original Class Label	Attacks Applied	Predicted Label After Attack	Accuracy with Class DR1 (%)	Accuracy with Class DR2 (%)	Accuracy with Class DR3 (%)
DR1	FGSM	DR1	99.83	0.11	0
DR2	FGSM	DR2	23.52	74.94	1.54
DR3	FGSM	DR3	2.89	0.02	97.09
DR1	SN	DR1	94.46	4.83	0.71
DR2	SN	DR2	0	99	1
DR3	SN	DR3	17.9	0	82.09
DR1	DF	DR1	96.51	0	3.29
DR2	DF	DR2	0	100	0
DR3	DF	DR3	0	0.01	99.99

**Table 7:** Summary of proposed defense model.

Training Dataset	Testing Dataset	Correct Label Prediction %
Original Dataset	Original dataset	100%
Original Dataset	FGSM Attacked Dataset	0%
Original Dataset	SN Attacked Dataset	10%
Original Dataset	DF Attacked Dataset	0%
Adversarial Training (AT1)	Original+ FGSM	62%
Adversarial Training (AT2)	Original+SN	52%
Adversarial Training (AT3)	Original+DF	66%
Adversarial Training (Mixed Data MAT)	Original+FGSM+SN+DF	92%

**Table 8:** Accuracy obtained using feature fusion approaches on different models.

Model	SVM	KNN (Cubic)	Ensemble
DarkNet-53	80.9%	79.6%	90.3%
HOG+SFTA+LBP	82.3%	84.1%	85.5%
Proposed Model	99.9%	99.5%	99.9%

**Table 9:** Results obtained using feature fusion approaches on different target classes.

Class	No of Instances	Accuracy (%)	Precision	Recall	F1-Score
DR1	2543	99.94	1.0	1.0	1.0
DR2	2509	99.95	1.0	1.0	1.0
DR3	1591	99.98	1.0	1.0	1.0

## Data Availability

Not applicable.
